# Presence of diabetes autoantigens in extracellular vesicles derived from human islets

**DOI:** 10.1038/s41598-017-04977-y

**Published:** 2017-07-10

**Authors:** Craig P. Hasilo, Sarita Negi, Isabelle Allaeys, Nathalie Cloutier, Alissa K. Rutman, Marco Gasparrini, Éric Bonneil, Pierre Thibault, Éric Boilard, Steven Paraskevas

**Affiliations:** 1 Human Islet Transplant Laboratory, McGill University Health Centre, Montréal, Québec Canada; 2 Research Institute of the McGill University Health Centre, Montréal, Québec Canada; 3 Centre de Recherche en Rhumatologie et Immunologie, Centre de Recherche du Centre Hospitalier Universitaire de Québec, Faculté de Médecine de l’Université Laval, Québec, Québec Canada; 4 Institut de Recherche en Immunologie et en Cancérologie, Université de Montréal, Montréal, Québec Canada; 5 Canadian National Transplant Research Program, Edmonton, Alberta Canada

## Abstract

Beta-cell (β-cell) injury is the hallmark of autoimmune diabetes. However, the mechanisms by which autoreactive responses are generated in susceptible individuals are not well understood. Extracellular vesicles (EV) are produced by mammalian cells under normal and stressed physiological states. They are an important part of cellular communication, and may serve a role in antigen processing and presentation. We hypothesized that isolated human islets in culture produce EV that contain diabetes autoantigens (DAA) from these otherwise normal, non-diabetic donors. Here we report the caspase-independent production of EV by human islets in culture, and the characterization of DAA glutamic acid decarboxylase 65 (GAD65) and zinc transporter 8 (ZnT8), as well as the β-cell resident glucose transporter 2 (Glut2), present within the EV.

## Introduction

An underlying hallmark of type 1 diabetes (T1DM) is the direct injury to the insulin-producing β-cells within the islets of Langerhans. Associated with a break in self-tolerance in these individuals, is the development of diabetes-associated autoantibodies (AAb). The major AAb found to correlate with diabetes progression (at least one AAb is detected in 90% of patients^1^) include those against the 65 kDa form of glutamic acid decarboxylase (GAD65)^2^, the zinc transporter 8 (ZnT8)^3^, the tyrosine phosphatase islet antigen-2 (IA-2)^4^, and insulin/proinsulin^5^. The detection of at least two AAb in asymptomatic individuals is defined as presymptomatic normoglycemia, a state which carries 5-year and 10-year risks of 44 and 70%, respectively, in progressing to overt diabetes^6^. Although the development of insulitis, β-cell injury, and AAb have been extensively studied, the factors or signals which initiate this process are not known.

Extracellular vesicles (EV) are produced by most, if not all cells under various stimuli. It is widely accepted that EV are an important part of cellular communication, and may play a role in the development of immune responses. Indeed, a pathophysiological role for EV have been identified in disease states such as cancer^7, 8^, rheumatoid arthritis^9^, systemic lupus erythematosis^10^, thrombocytopenia^11^, as well as neurodegenerative^12^ and vascular disease^13, 14^, to name a few. The cargo payload carried by EV varies greatly depending on their size and origin. Traditionally, EV have been classified according to size, with exosomes (Exo) generally representing EV of 150 nm or less, microvesicles (MV) being 100 to 1,000 nm, and apoptotic bodies (AB) ranging from 1,000 to 5,000 nm and often containing nucleic acids, mitochondrial proteins and other subcellular structures^15^. More recently, however, it has been illustrated that the more encompassing term EV should be used, as it accurately reflects all vesicle types released by cells^16^, as Exo up to 250 nm and Exo-like vesicles derived from apoptotic cells have been described^13, 17^. EV may contain neo-antigens (Ag) that, when exposed in times of injury, may act as triggers of autoimmunity^13^.

EV production in the context of T1DM is only beginning to be appreciated with a few preliminary reports in murine and human contexts. Exo from mouse insulinoma cell lines or islet-derived mesenchymal stem cells have immunostimulatory capacity in non-obese diabetic (NOD) mice, eliciting a response in autoreactive T-cells and marginal zone-like B-cells^18–20^. Human islets have been evaluated for miRNA content in the context of islet-endothelial cell interactions^21^, and produce GAD65, IA-2 and proinsulin containing-Exo, following cytokine exposure^22^.

**Figure 1:**
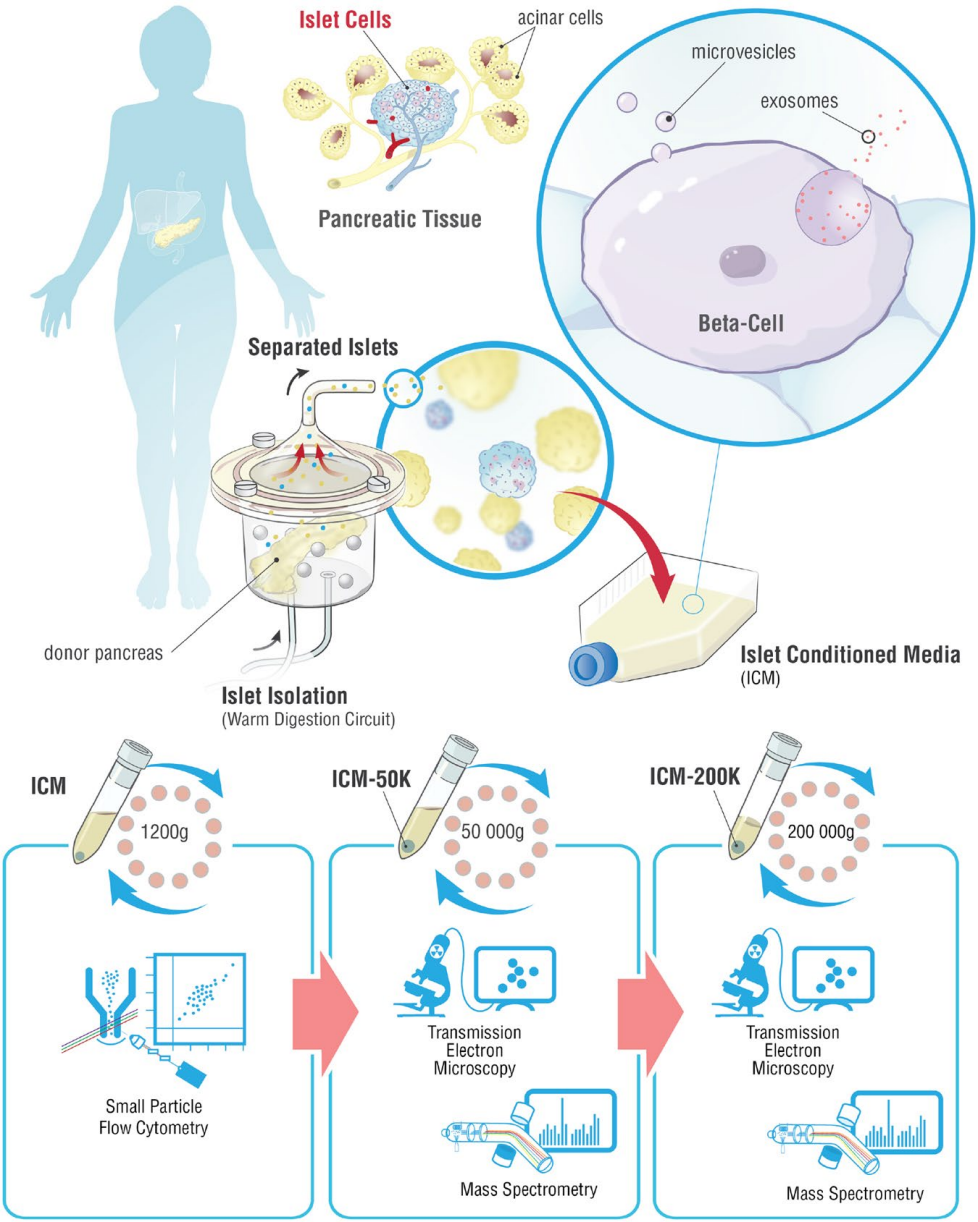
Schema describing the workflow to generate and analyze human islet conditioned media extracellular vesicles. Islet isolation was performed after the surgical retrieval of a donor pancreas. The pancreas was enzymatically digested in a closed loop circuit until free islets were observed. The islets were then purified, and cultured for up to 72 hours. Islet conditioned medium (ICM) was harvested, centrifuged at 1,200 × g for 15 min. and analyzed by small particle flow cytometry for DAA and markers of EV, or serially centrifuged a 50,000 × g and 200,000 × g for analysis by transmission electron microscopy or mass spectrometry on the ICM-50 K and ICM-200 K fractions.

Our study focused on determining whether human islet-derived EV harbor known diabetes autoantigens (DAA). The possibility exists that some β-cell components not normally externalized may be released following sub-lethal injury^23^. Here we report the production of EV by normal, nondiabetic human islets following isolation, their characterization using flow cytometry and proteomic-based approaches, and the observation of T1DM autoantigens in these extracellular particles (Fig. 1).

## Results

### Human islets produce a heterogeneous EV population in culture

**Figure 2:**
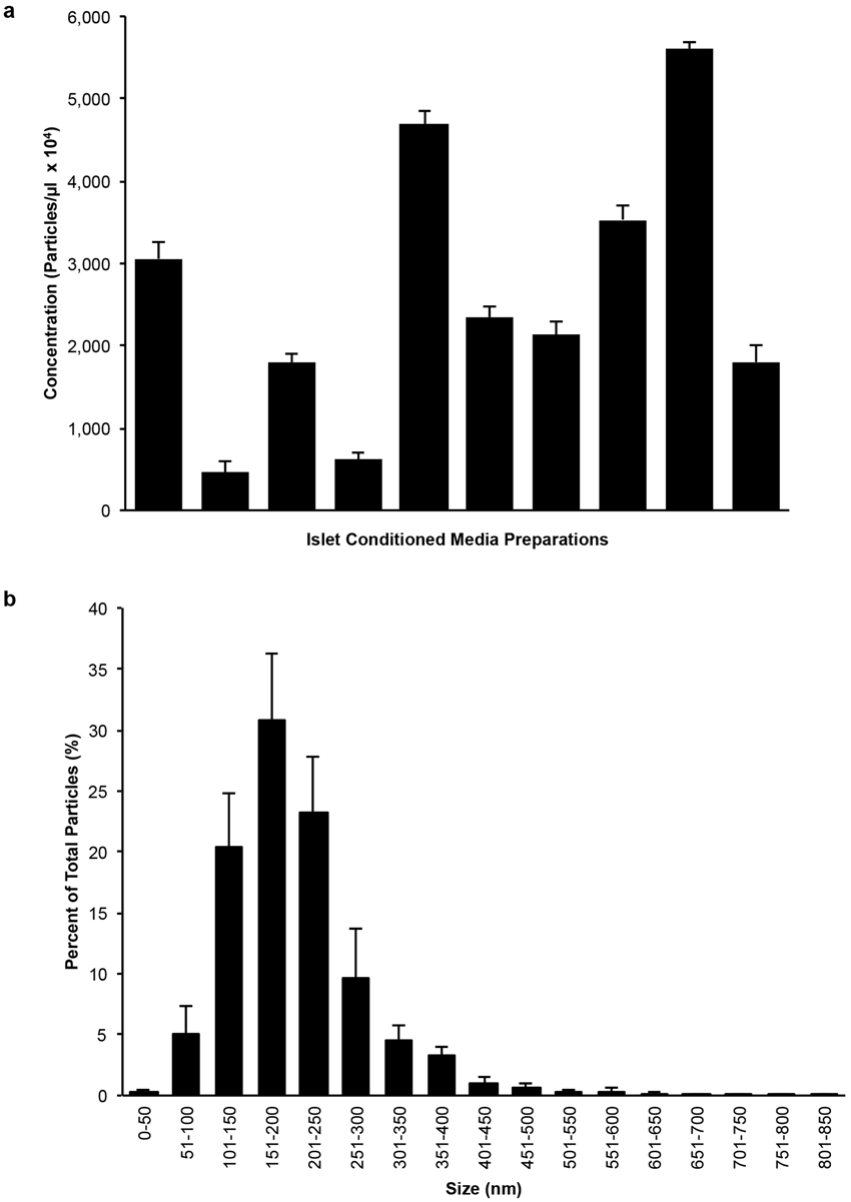
Cultured human islets produce distinct populations of particles. (**a**) Nanoparticle tracking analysis (NTA) graph of mean particle levels in ICM from ten donors (n = 10) shows variable levels between preparations. (**b**) NTA histogram of particle size distribution shows the majority of particles detected are between 100 nm and 650 nm. Data are expressed as mean ± SEM of ten donors (n = 10). At least 5 recordings, 30 seconds each were obtained for each sample at 37 °C, camera shutter speed of 30.0 ms, camera level 14 and detection threshold set to 9.

To evaluate if our human islet preparations were producing EV in culture, we first used Nanoparticle Tracking Analysis (NTA) to quantify the release of particles by islet preparations. Since it has been suggested that culture conditions may result in varying levels of EV secreted into the media, we first investigated EV elution after 24 hours. Particle tracking by NTA revealed concentrations that varied between donor islet preparations. Generally, the islet preparations produced between 4.53 ± 1.49 × 10^6^ to 5.61 ± 0.88 × 10^7^ particles/µl (Fig. 2a), with a mean size of 193 ± 21 nm (Fig. 2b).

**Figure 3:**
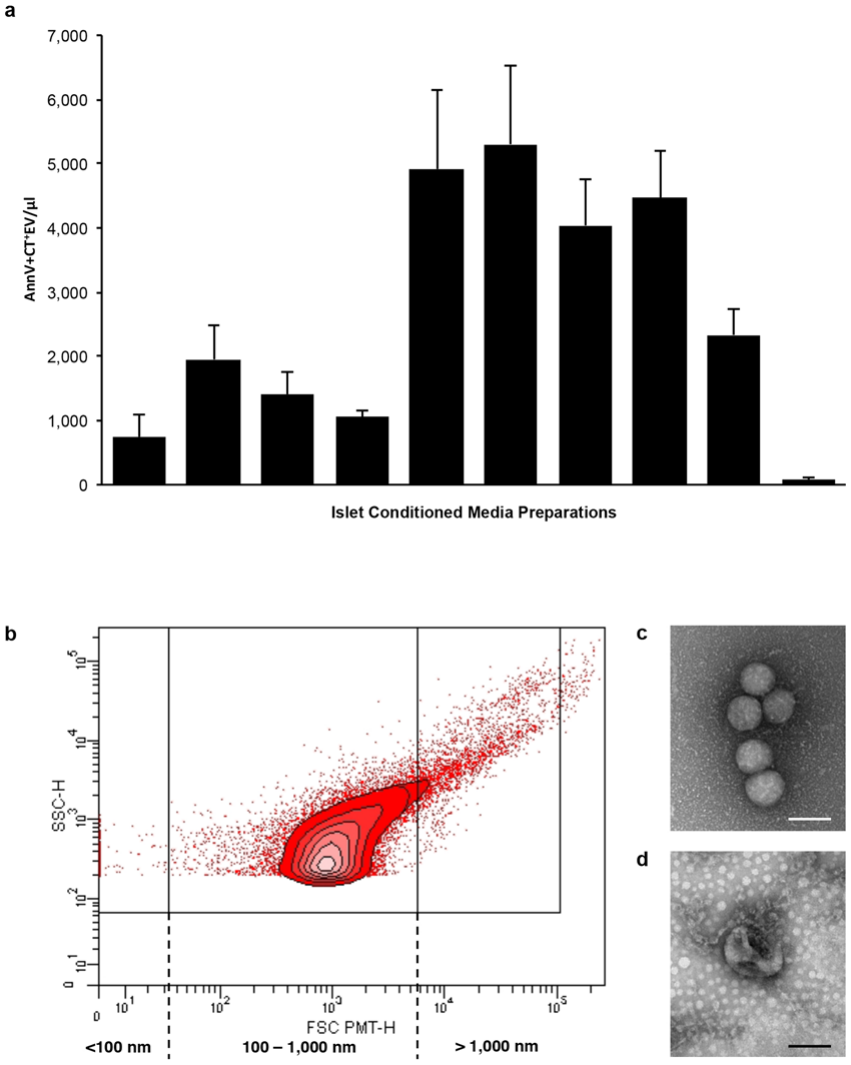
Human islet conditioned media contains a heterogeneous population of AnnV^+^CellTracker^+^EV. (**a**) Bar graph showing the mean levels of each ICM (n = 10) detected by flow cytometry varies in their content of AnnV^+^Cell Tracker^+^EV after 24 hours in culture. (**b**) Representative contour plot (FSC vs SSC) shows the peak density of AnnV^+^Cell Tracker^+^EV is within 100 nm to 1,000 nm size based on the detection of 100, 500 and 1,000 nm silica beads. (**c**,**d**) Visualization of fractionated ICM by whole mount negative staining TEM confirms the presence of membrane vesicles. Scale bars represent 100 nm. Data are expressed as mean ± SEM of n = 3 independent experiments performed on ICM from ten (n = 10) different donors.

To further characterize EV and to confirm their membrane moiety, we analyzed islet conditioned media (ICM) from each islet preparation by flow cytometry. Annexin V (AnnV) and CellTracker Deep Red (CT) were respectively chosen as markers of membrane-phospholipid moiety (phosphatidylserine, PS) and of cytosol content, as in previous studies^9, 13, 24–26^. Similar variability was observed by flow cytometry analyses, as EV were detected in quantities ranging from 70.77 ± 44.86 to 5,302.36 ± 1,236.71 AnnV^+^CT^+^EV/µl between ICM preparations (Fig. 3a) containing a heterogeneous population of AnnV^+^CT^+^EV with a peak density in the 100–1,000 nm range (Fig. 3b). The addition of the detergent Triton X-100 to the ICM resulted in a dose-dependent decrease in AnnV^+^EV (Supplementary Fig. S1), confirming the signal detected was indeed due to the presence of membrane bound vesicles. We then performed whole mount negative staining with transmission electron microscopy (TEM). Visualization of the fractionated ICM confirmed the presence of membrane vesicles contained within (Fig. 3c,d).

### Proteomic analysis of islet-derived EV shows commonality with that of islet lysates

**Figure 4:**
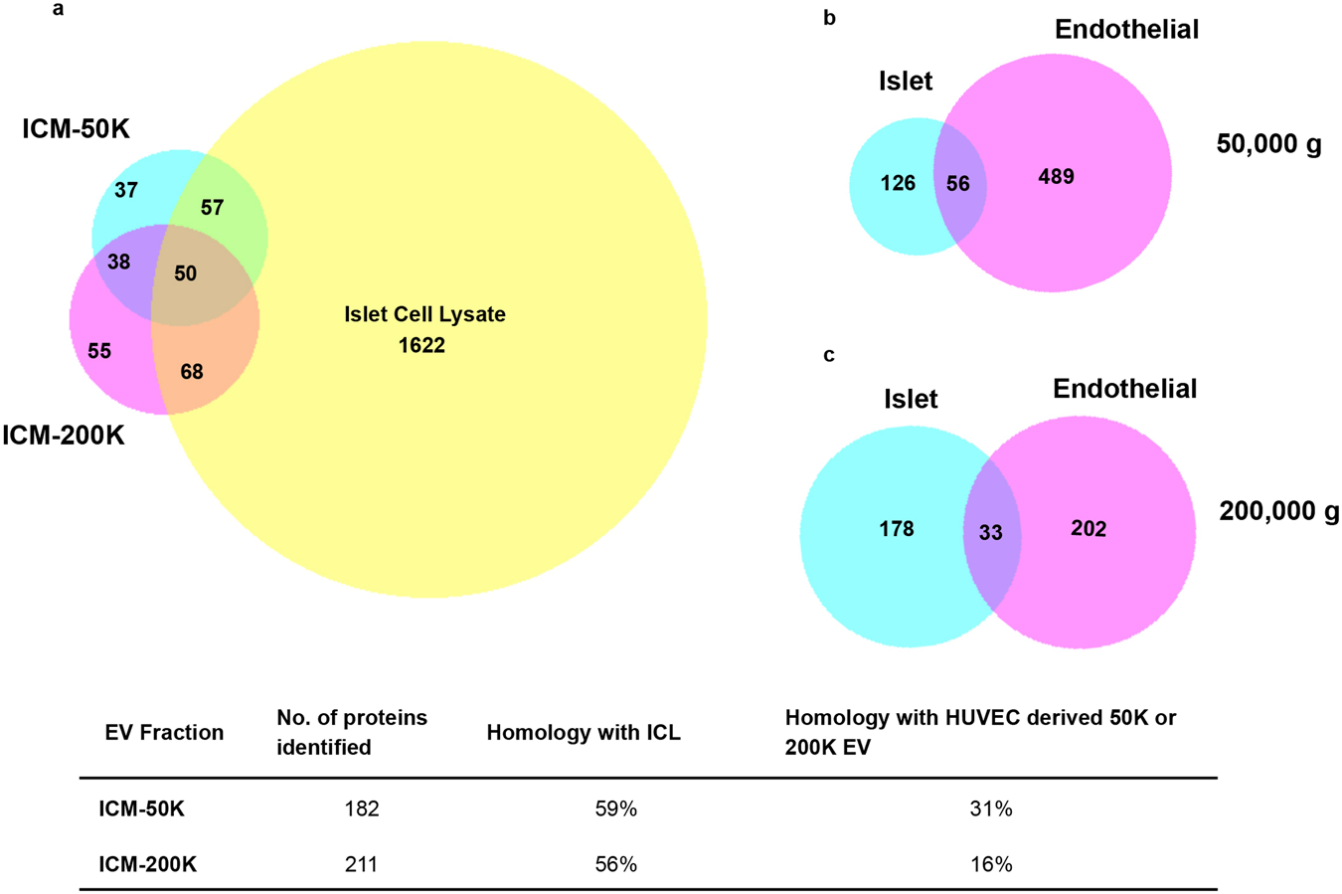
Proteomic analysis reveals homology of islet conditioned media extracellular vesicles with islet cell lysate, and a signature distinct from extracellular vesicles produced by human umbilical vein endothelial cells. Venn diagrams depicting the overlap of genes detected in the islet conditioned media (ICM)-50 K and ICM-200 K fractions with that of the islet cell lysate (ICL) (**a**). Comparison of homology of genes detected in ICM-50 K compared to that of human umbilical vein endothelial cell (HUVEC)-50 K, as well as in ICM-200 K and HUVEC-200 K (islet and endothelial 50,000 g (**b**) and 200,000 g (**c**), respectively).

In order to obtain a complete protein profile of vesicles from ICM, protein extracts from 50,000 × g (50 K) and 200,000 × g (200 K) serial centrifugations of ICM (ICM-50 K and ICM-200 K, respectively), and islet cell lysates (ICL) were used for analysis by liquid chromatography-mass spectrometry (LC-MS/MS). We identified 182, 211, and 1,622 proteins in ICM-50 K, ICM-200 K, and ICL, respectively (Fig. 4a). Interestingly, all the fractions from ICM showed ≥56% homology with ICL indicating that islet-derived vesicles carry islet-specific proteins. To confirm that this data set is unique to islets, we compared our data to the proteins identified in the serially centrifuged 50 K and 200 K fractions generated from conditioned media harvested from a serum-starved human umbilical vein endothelial cell line (HUVEC; ExoCarta and Vesiclepedia databases, unique accession ID: vesiclepedia_560). Indeed, the commonality between the human ICM-50 K and ICM-200 K fractions compared to that of the HUVEC were 31 and 16%, respectively (Fig. 4b,c). These observations suggest that islet-derived EV carry cell-type specific protein signatures. The proteomic data will be made available by submission to ExoCarta and Vesiclepedia.

**Figure 5:**
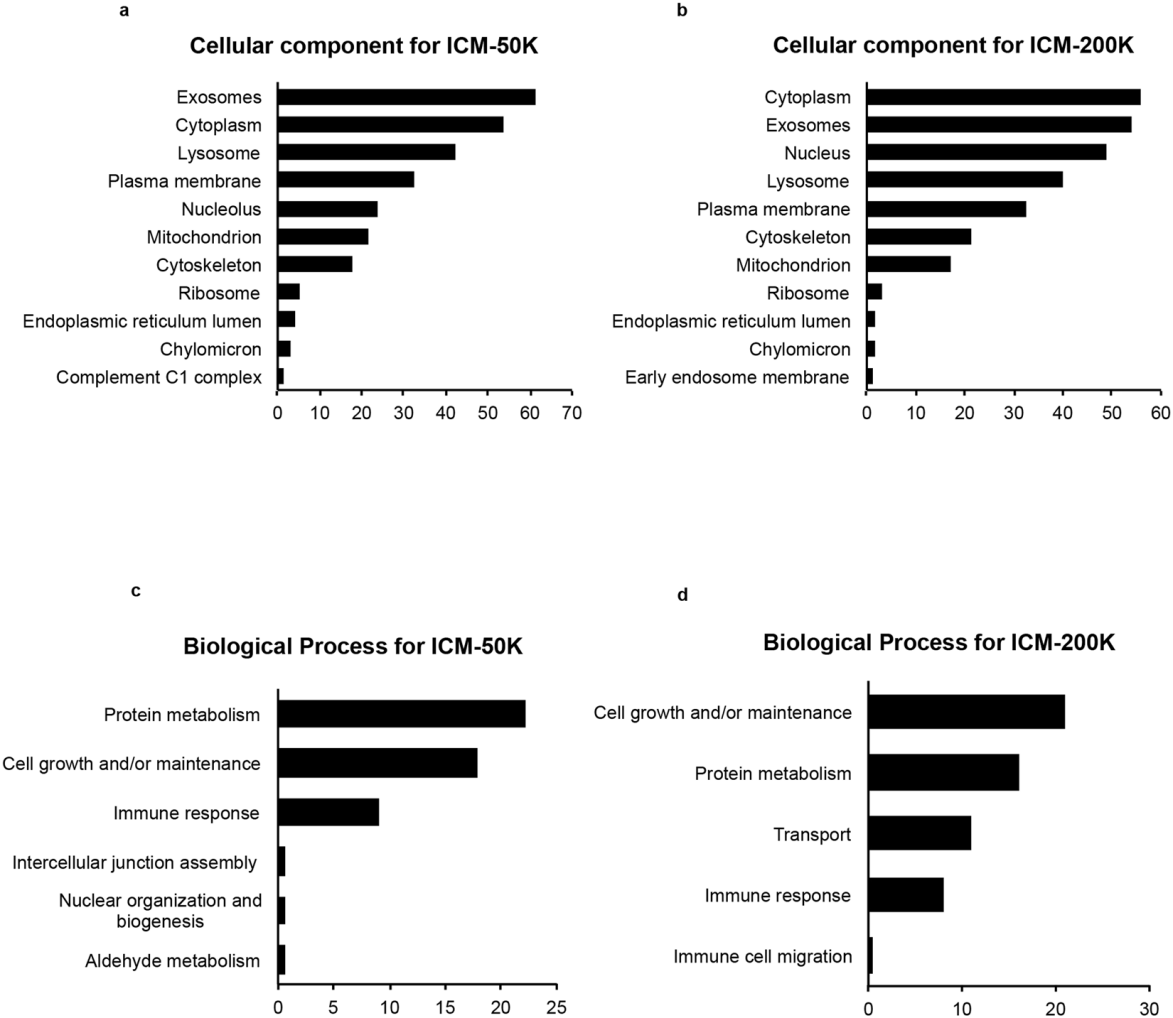
Comparison of proteomic data by enrichment analyses of fractionated human islet conditioned media with FunRich component and pathway analyses. Bar graphs of cellular compontments overrepresented in highly abundant proteins are shown for islet conditioned media (ICM)-50 K (**a**) and ICM-200 K (**b**). Bar graphs illustrating the biological process overrepresented in highly abundant proteins for ICM-50 K (**c**) and ICM-200 K (**d**).

We performed the Go-term enrichment analysis to evaluate the distribution of identified proteins by putative cellular compartment, biological process and molecular function. Enrichment of cellular component showed that a large number of proteins present in islet derived EV are of exosomal origin (Fig. 5a,b) and carry several proteins commonly associated with EV, such as annexins, heat shock protein 70 kDa and 90 kDa variants (Hsp70 and Hsp90, respectively), integrins, clathrin, Ras-related protein Rab5C, and cluster of differentiation factor 14 (CD14) to name a few (Table 1). The majority of the identified proteins in these vesicles were associated with cytoplasm, membrane and cytoskeleton (Fig. 5a,b). Islet-derived EVs were also enriched in proteins associated with lysosomes, mitochondria and nucleolus (Fig. 5a,b). Moreover, enrichment analysis for molecular function identified proteins involved in immune response, heat shock and complement activation were also enriched in our EV (Fig. 5a,c,d, Supplementary Fig. S2a,b).

**Table 1:** List of proteins commonly associated with extracellular vesicles and islet cells identified by LC-MS/MS within fractionated human ICM.

Accession No.	Symbol	Name	ICM-EV fraction
P07355	ANXA2	Annexin A2	ICM-200 K
P09525	ANXA4	Annexin A4	ICM-50 K
P08758	ANXA5	Annexin A5	ICM-50 K
P08133	ANXA6	Annexin A6	ICM-50 K
P20073	ANXA7	Annexin A7	ICM-50 K
P14625	HSP90B1	Endoplasmin	ICM-50 K, ICM-200 K
P34932	HSPA4	Heat shock 70 kDa protein 4	ICM-200 K
P11021	HSPA5	78 kDa glucose-regulated protein	ICM-50 K
P11142	HSPA8	Heat shock cognate 71 kDa protein	ICM-50 K, ICM-200 K
P38646	HSPA9	Stress-70 protein, mitochondrial	ICM-50 K
P04792	HSPB1	Heat shock protein beta-1	ICM-200 K
P04406	GAPDH	Glyceraldehyde-3-phosphate dehydrogenase	ICM-200 K
P60709	ACTB	Actin, beta	ICM-200 K
P06733	ENO1	Enolase 1, (alpha)	ICM-200 K
P14618	PKM	Pyruvate kinase, muscle	ICM-50 K, ICM-200 K
P68104	EEF1A1	Eukaryotic translation elongation factor 1 alpha 1	ICM-50 K, ICM-200 K
P04075	ALDOA	Aldolase A, fructose-bisphosphate	ICM-50 K
P13639	EEF2	Eukaryotic translation elongation factor 2	ICM-200 K
P00558	PGK1	Phosphoglycerate kinase 1	ICM-50 K
P62258	YWHAE	Tyrosine 3-monooxygenase/tryptophan 5-monooxygenase activation protein, epsilon	ICM-50 K
Q00610	CLTC	Clathrin, heavy chain (Hc)	ICM-200 K
P62937	PPIA	Peptidylprolyl isomerase A (cyclophilin A)	ICM-50 K
P60174	TPI1	Triosephosphate isomerase 1	ICM-50 K
Q06830	PRDX1	Peroxiredoxin 1	ICM-200 K
P21333	FLNA	Filamin A, alpha	ICM-50 K, ICM-200 K
P01023	A2M	Alpha-2-macroglobulin	ICM-50 K, ICM-200 K
P51148	RAB5C	RAB5C, member RAS oncogene family	ICM-200 K
P01275	GCG	Glucagon	ICM-50 K, ICM-200 K
P08571	CD14	CD 14 molecule	ICM-50 K, ICM-200 K
P61278	SMS	Somatostatin	ICM-50 K, ICM-200 K

### DAA are present in the EV produced by cultured human islets

**Figure 6:**
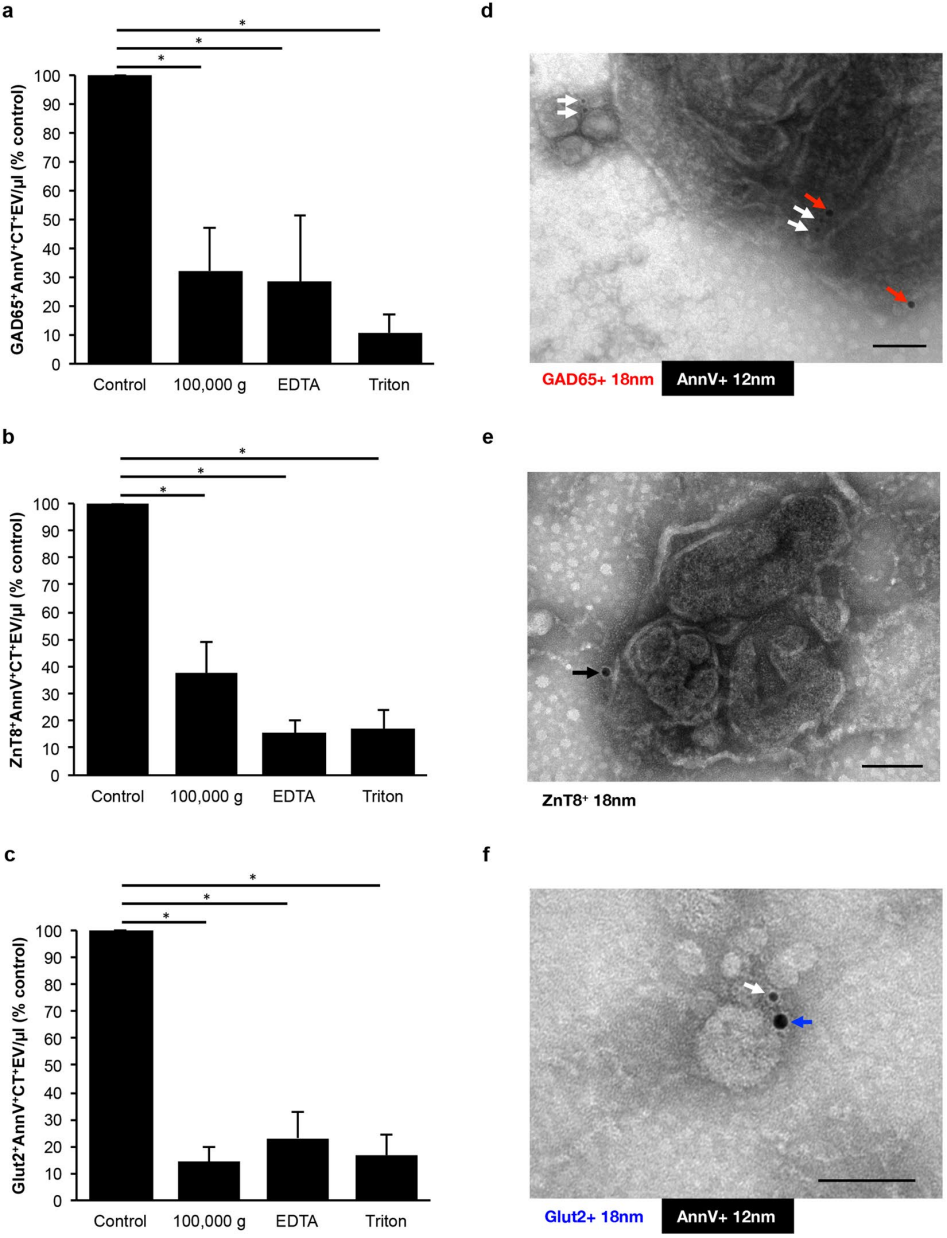
Diabetes autoantigens and β-cell markers are present on extracellular vesicles produced by non-diabetic donors. (**a**–**c**) Bar graphs of islet conditioned media (ICM) compared to signal depletion by 100,000 g centrifugation, 50 mM EDTA, or 0.3% Triton X-100 for GAD65^+^AnnV^+^CT^+^EV (**a**), ZnT8^+^AnnV^+^CT^+^EV (**b**), and Glut2^+^AnnV^+^CT^+^EV (**c**). (**d**–**f**) Micrographs of immunogold double labeled islet-derived EV preparations from fractionated ICM confirms the presence of AnnV (white arrows), GAD65 ((**b**) red arrows), ZnT8 ((**d**) black arrow) and Glut2 ((**f**) blue arrow). Data are expressed as mean ± SEM from n = 4 human islet preparations from different donors. *p < 0.05, repeated measures one-way analysis of variance with Tukey’s post-hoc test performed on raw values prior to conversion to percent of control. Scale bars represent 100 nm.

**Figure 7:**
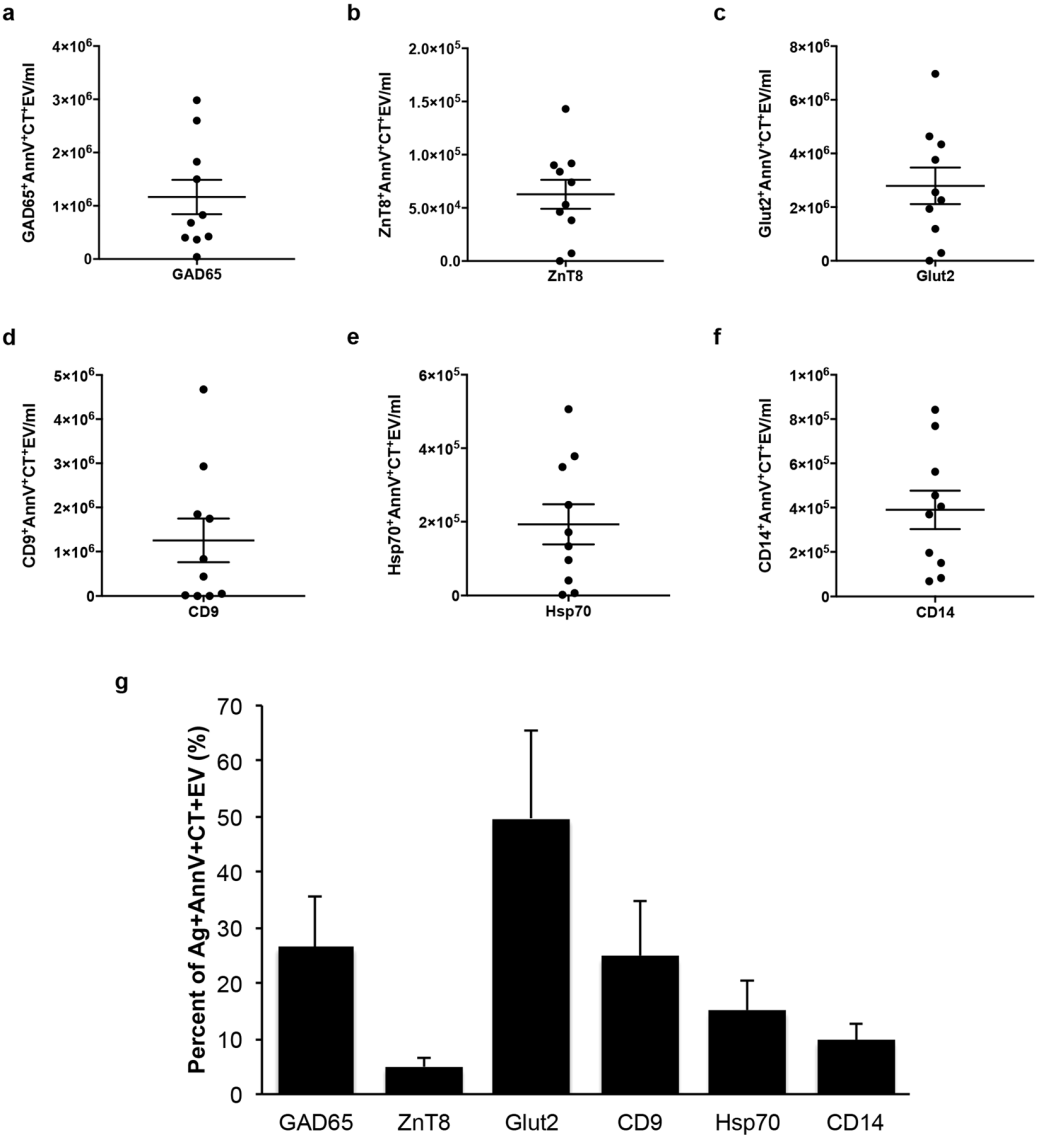
Antigen levels on extracellular vesicles present in islet conditioned media from different donor islet preparations. Column scatter graphs of islet conditioned media (ICM) analyzed by flow cytometry for GAD65^+^AnnV^+^CT^+^EV (**a**), ZnT8^+^AnnV^+^CT^+^EV (**b**), Glut2^+^AnnV^+^CT^+^EV (**c**), CD9^+^AnnV^+^CT^+^EV (**d**), Hsp70^+^AnnV^+^CT^+^EV (**e**), and CD14^+^AnnV^+^CT^+^EV (**f**). Bar graph depicting the percent of AnnV^+^CT^+^EV that were positive for the antigens described in the above panels (**g**). Data are expressed as mean ± SEM from n = 10 human islet preparations from different donors, and are representative of n = 3 independent experiments.

To investigate the presence of DAA in human ICM EV, we employed flow cytometry with a small particle analyzer, and gated on individual autoantigens in combination with the EV markers AnnV and CT. This analysis revealed the presence of Ag^+^AnnV^+^CT^+^EV for GAD65 (Fig. 6a), ZnT8 (Fig. 6b), the β-cell facilitative glucose transporter, Glut2 (Fig. 6c), the tetraspanin superfamily member, cluster of differentiation factor 9 (CD9) (Supplementary Fig. S3a), and confirmed the proteomic detection of Hsp70 (Supplementary Fig. S3b), and CD14 (Supplementary Fig. S3c), but not IA-2, nor heat shock cognate 70 kDa variant (Hsc70) in the ICM tested (Table 2). Individual contour plots for each of these biomarkers revealed a peak density of EV within a similar size range to that of AnnV^+^CT^+^EV alone (Supplementary Fig. S4). Similar to the variability seen with AnnV^+^CT^+^EV alone, we noted a wide variation in Ag^+^AnnV^+^CT^+^EV levels between islet preparations (Fig. 7a–f) and in the total percentage of AnnV^+^CT^+^EV with detected antigens on the surface (Fig. 7g). To ensure that genuine EV were being identified and to verify the specificity of the antibodies used, samples of ICM from different islet preparations (n = 4 donors) were treated with centrifugation at 100,000 × g for 1 hour, 50 mM ethylenediaminetetraacetic acid (EDTA), or 0.3% Triton X-100, with resulting signal depletion. In the case of GAD65, a significantly greater level of GAD65^+^AnnV^+^CT^+^EV/µl was observed between control (non-treated ICM) and ICM centrifuged at 100,000 × g (p = 0.012), ICM with 50 mM EDTA (p = 0.005) and ICM treated with 0.3% Triton X-100 (p = 0.005), indicating that the markers detected did indeed come from the EV present within the ICM (Fig. 6a and Supplementary Fig. S4). The same pattern was observed for each of ZnT8 (Fig. 6b), Glut2 (Fig. 6c), CD9 (Supplementary Fig. S3a), Hsp70 (Supplementary Fig. S3b), and CD14 (Supplementary Fig. S3c), where treatment of the ICM by 100,000 × g centrifugation, EDTA and Triton X-100 all resulted in a significant depletion of Ag^+^AnnV^+^CT^+^EV/µl detected (p < 0.05).

**Table 2:** List of autoantigens and biomarkers assessed, and the method used for their detection, within the human islet conditioned media.

Marker	Category	Description	Detection
GAD65 (GAD2)	Diabetes autoantigen	Diabetes autoantigen; enzyme that converts glutamate to GABA for storage in secretory vesicles in pancreatic β-cells and GABA-secreting neurons^41^.	Flow Cytometry, TEM
IA-2 (PTPRN)	Diabetes autoantigen	Islet antigen-2; protein tyrosine phosphatase family (PTPRN); enriched in pancreatic β-cells. Membrane bound associated with insulin granule^5, 64^.	Not detected
ZnT8 (SLC30A8)	Diabetes autoantigen	Zinc transporter present on the cell surface and localized to the insulin storage granules. Highest levels are within the pancreatic β-cell^45^.	Flow Cytometry, TEM
Glut2 (SLC2A2)	β-cell marker; New-onset diabetes autoantigen	Glucose transporter-2; resident on cell surface for passive diffusion of glucose into cell. High levels on pancreatic β-cells, hepatocytes, kidney proximal tubule cells and cells within the small intestine^49^.	Flow Cytometry, TEM
Hsc70	β-cell marker	Heat shock cognate protein 70, membrane associated, maintains GAD65 in close proximity to GABA-containing vesicles^65^, common EV marker.	LC-MS/MS
Hsp70	Injury marker	Highly stress-inducible injury marker, stabilizes proteins against aggregation, known to be pro-inflammatory^66^, common marker of EV.	Flow Cytometry, LC-MS/MS
CD9	EV marker	Tetraspanin, four transmembrane spanning regions involved in protein scaffold maintenance, modulation of cell adhesion, motility and migration, membrane fusion, and exocytic vesicle formation^67^.	Flow Cytometry
CD14	EV Marker	Monocyte differentiation antigen. Acts as a GPI-anchored coreceptor for TLR2:TLR6; internalized into the endosomal compartment in a lipid raft-dependent mechanism^68^.	Flow Cytometry, LC-MS/MS
Annexin V (ANXA5)	EV marker	Intracellular protein with anchoring domains that binds to calcium and to phospatidylserine^69^.	Flow Cytometry, LC-MS/MS, TEM

TEM analyses revealed a population of EV ranging in size from 30 to 700 nm (Figs. 3c,d and 6d–f, and Supplementary Fig. S5). Assessment of immunogold double labeling on EV by whole mount negative staining TEM revealed Ag^+^AnnV^+^, Ag^+^AnnV^−^, Ag^−^AnnV^+^, and Ag^−^AnnV^−^EV for DAA GAD65 (Fig. 6d and Supplementary Fig. S5a,d) and ZnT8 (Fig. 6e and Supplementary Fig. S5b,e), and β-cell marker Glut2 (Fig. 6f and Supplementary Fig. S5c,f).

To assess the contribution of caspase-dependent apoptosis to islet-derived EV, we cultured islet preparations (n = 3 donors) with 50 μM of the pan-caspase inhibitor carbobenzoxy-valyl-alanyl-aspartyl-[O-methyl]-fluoromethylketone (ZVAD) or dimethyl sulfoxide (DMSO; vehicle) and compared EV production to that of islets in culture with no intervention (Normal). No difference was observed on GAD65^+^AnnV^+^CT^+^EV (Supplementary Fig. S6a) or AnnV^+^CT^+^EV (Supplementary Fig. S6b) production in the ICM between ZVAD, DMSO and Normal islets (p > 0.05). A time course assessment of EV production revealed that the greatest concentration of both GAD65^+^AnnV^+^CT^+^EV and AnnV^+^CT^+^EV were produced at 24 hours (p < 0.05), declined at 48 hours and then stabilized by 72 hours, but this was independent of ZVAD treatment (Supplementary Fig. S6). Furthermore, there was no size-dependent treatment effect observed on EV production (Supplementary Fig. S7).

## Discussion

In this study, we have demonstrated the production of a heterogeneous population of EV by islets in culture, and characterized human islet-derived EV with respect to DAA and β-cell markers. These EV expose PS on their outer surface, as assessed by AnnV binding, contain intracellular esterases of islet-origin, as assessed using CT, and present DAA GAD65 and ZnT8, and glucose transporter Glut2. We have shown the precise detection of each of these Ag-containing vesicles using flow cytometry, as centrifugation at 100,000 g, the addition of EDTA to chelate free calcium and prevent AnnV binding to PS, and the detergent-based depletion of membrane bound structures via the addition of Triton X-100 all significantly reduced the detection of Ag positive EV. Visualization of these DAA-containing EV was confirmed by whole mount immunogold TEM, showing a morphological diversity of EV in human ICM. A large proportion of vesicles were AnnV negative, as has been reported in the case of platelet-derived microparticles^27, 28^ and adipocyte-derived EV^29^. Vesicles that are PS negative may represent a population of EV that are less immunostimulatory and capable of remaining in the circulation for longer durations. The significance of AnnV negative particles is not yet fully understood, and while they may represent an underappreciated population of EV warranting further investigation^30^, analyses of these EV falls out of the scope of this present study.

While the concentration of EV released into the ICM varied dramatically between islet preparations, as detected by NTA (Fig. 2a) and flow cytometry (Fig. 3a), the majority of the ICM contained EV that were consistent in the profile of size distribution. The discrepancy may be due to the fact that for flow cytometry, we conservatively evaluated EV harboring PS and containing esterases, which are respectively indicators of membrane moiety and cytosol content, whereas NTA counts EV without regard to specific markers. Notably, the particle size distribution was similar between NTA and flow cytometry with a range of 100 to 1,000 nm (Figs 2b and 3b). Furthermore, the visualization of islet-derived EV by whole mount negative staining TEM confirmed that the islet-derived EV were indeed membrane-bound structures (Figs 3c,d and 6d–f and Supplementary Fig. S5). Other groups have similarly reported that the majority of protocols used to isolate EV (or more specifically, Exo) result in diverse populations of EV from endosomal and non-endosomal origin^15, 16, 31^.

Proteomic evaluation of islet-derived EV reflected an islet-specific protein signature as they shared ≥56% homology with human islets but not with endothelial cell-derived vesicles (Fig. 4). Several common markers of EV were detected in both our ICM-50 K and ICM-200 K by LC-MS/MS, such as members of the annexin family, heat shock proteins, clathrin, Rab5C and CD14 (Table 1), confirming that the vesicles detected did in fact contain proteins corresponding to transmembrane or lipid-bound, cytosolic and intracellular compartmental origins (Fig. 5a,b). The presence of proteins from mitochondria and lysosomes suggest that the islets might be undergoing autophagy and cell death, as has been reported after the mechanical and physical stress during the isolation procedure^32, 33^. Apoptotic Exo-like vesicles from ischemic or stressed cells are known to stimulate autoantibody production, as is the case for the serum-starved HUVEC used to compare with that of our fractionated ICM EV data set^13^. The EV we have detected in human ICM may also contribute to the stimulation of an inflammatory response, as they carry proteins involved in apoptotic pathways, chaperone or complement activation (Fig. 5, Supplementary Fig. S2). This could further pose a problem for regenerative cellular therapies to treat T1DM, such as islet transplantation, as a re-appearance or increase in AAb may be related to β-cell failure post-transplant^34^. Overall, these observations demonstrate that an islet-specific protein signature coexists with the proteins associated with immune response on islet derived EV, suggesting their potential to promote an islet-specific immune response.

A recent study confirms our hypothesis that DAA are contained within EV produced by human islets. This study documents the trafficking of GAD65, IA-2 and proinsulin in Exo produced from the *trans*-golgi network in a Rab11-controlled endosomal pathway by humans and rat islets in culture^22^. These DAA containing Exo were taken up by activated dendritic cells, with a concurrent cytokine-induced increase in production of Exo and uptake by dendritic cells under stimulation, thus detailing a potential antigen-presentation model for the break in self-tolerance in T1D. In this study, the authors were able to detect GAD65 and IA-2 in their EV preparations by LC-MS/MS by introducing a pre-purification of their proteomic samples with an additional SDS-PAGE step prior to tryptic digestion. In our study, the presence of albumin in the culture media necessitated that albumin-containing bands be cut out prior to tryptic digestion. In this way, proteins of similar size to the 66.5 kDa albumin may have been excised with the albumin-containing band and therefore not included in the tryptic digests. While this may account for GAD65 not being detected in our LC-MS/MS analyses, other factors may be at play.

A well-documented limitation of LC-MS/MS is the inefficient solubilization of transmembrane and membrane-anchored proteins due to their hydrophobicity and poor ionization of peptides^35–37^. Tryptic digestion is not always sufficient to release peptide fragments contained within the transmembrane regions, hindering the detection of these markers. In the case of GAD65, the NH_2_-terminal membrane anchoring region has been identified as being responsible for the hydrophobicity of the enzyme^38^. Similarly, ZnT8 belongs to the SLC family of zinc transporters that are predicted to contain 6 transmembrane domains^39^, and Glut2 belongs to the SLC2 family of membrane transporters containing 12 membrane-spanning domains^40^. This may explain why GAD65, ZnT8 and Glut2 were not detected in our LC-MS/MS analyses, despite their identification by flow cytometry and immunogold TEM. The same may hold true for CD9, CD81 and other members of the tetraspanin family that are often detected as common EV markers.

In its physiological state, GAD65 has been localized within β-cells to subcellular MV and Golgi membranes^41, 42^. An accumulation of GAD65 in β-cell Golgi membranes was shown in pancreas biopsies from T1DM patients and *in vitro* models of endoplasmic reticulum stress^43^. Palmitoylation of GAD65 was the cause of this accumulation and also the proposed mechanism for its immunogenicity once externalized.

The localization of ZnT8 to EV is an intuitive discovery. The storage of insulin within the dense core secretory vesicles necessitates the internalization of high concentrations of zinc for solid hexamer formation of 6 insulin units with two Zn^2+^ ions^44^. ZnT8 is expressed in the β-cells of human islets to orchestrate the zinc accumulation and colocalizes with the insulin granules^45^. The presence of ZnT8 on ICM EV could therefore represent products of insulin secretory granule reprocessing, and may indicate the degree of stress to the islets, as it was not detected on all ICM preparations. An association with disease risk has been reported for an epitope of ZnT8 that leads to an increased incidence of T1DM and type 2 diabetes mellitus (T2DM) in susceptible individuals. Variant loci with a common non-synonymous single nucleotide polymorphism have been identified in a susceptible population, which influence ZnT8 autoantibody specificity in T1DM^3, 46^, and also confer a greater risk of developing T2DM^47^. To the best of our knowledge, this represents the first report of a plausible mechanism of ZnT8 Ag delivery to Ag presenting cells.

Glut2 is a facilitative glucose transporter with tissue specificity for hepatocytes, the basolateral membranes of epithelial cells in the kidney proximal tubules, and enterocytes of the small intestines, and at high levels in the pancreatic β-cells^48^. Although it is not entirely specific to the pancreatic islet β-cells *in vivo*, AAb to Glut2 were previously reported in 77% of the new-onset T1DM patients screened, compared to a lack of anti-Glut2 antibody detection in 94% of non-diabetic control subjects^49^. This would suggest that anti-Glut2 antibodies are indicative of damage to pancreatic islets that still contain a population of intact β-cells and may thus be considered a marker of active β-cell destruction. In our model, the detection of Glut2 on the surface of eluted EV may be due to the injury sustained by the islets.

The stress or injury to human islets imposed by the isolation procedure has been well documented in the literature by our group and others. Ischemic stress is induced in all organs recovered from human multi-organ donors, even with relatively short periods of cold preservation^50, 51^. During the human islet isolation, stress from ischemia during the pancreas retrieval surgery and transport to the isolation laboratory^52^, warm ischemic injury during pancreatic enzymatic dissociation, and gradient purification prior to cell culture^53–56^, have all been attributed the induction of apoptosis in freshly isolated islets^57–59^. Introducing a period of recovery during islet culture is one method to reduce this stress, however, our focus in the current study was to determine how this stress may lead to the production of DAA-containing EV. One of the main goals of our study was to generate EV-based markers, specific to islet preparations that may be useful in clinical decision making. Therefore the onus on minimal sample manipulation was of the utmost importance.

It is interesting to note that the findings of Cianciaruso *et al*.^22^ did not include the detection of ZnT8 or Glut2. This may be due to several factors including the size-restricted analyses performed on Exo, whereby ZnT8 and Glut2 were localized to Exo-like and larger EV in our study (Fig. 6d–f and Supplementary Fig. S5). Furthermore, in that study, islets were allowed to recover for an unspecified period of time, then stimulated by cytokine administration. It is not known if this aspect may change the type and content of EV produced.

Taken together, the presence of the combination of DAA GAD65, ZnT8 and Glut2 on ICM EV suggest a novel, β-cell specific method of antigen presentation that is only beginning to be appreciated as a plausible contribution to the events that lead to a break in self-tolerance. The combination of GAD65, ZnT8 and Glut2 on islet-derived EV may be used as biomarkers for the detection of early islet injury and may serve to identify susceptible individuals for disease progression prior to AAb production.

## Methods

### Consent for research and regulatory approvals

This study was approved by the Centre for Applied Ethics at the McGill University Health Centre (MUHC). All studies were conducted in accordance with relevant guidelines and procedures on pancreatic islets originating from end-of-life organ donation after research consent was obtained from next-of-kin by the appropriate organ donation personnel at Transplant Québec. Only post-mortem biological samples were used in this study with no patient identifiers.

### Human islet isolation and the collection of islet conditioned media

Islets of Langerhans were isolated, purified, and cultured at the MUHC Human Islet Transplant Laboratory, using previously described protocols^60, 61^. Briefly, the pancreas was loaded intraductally with cold CIzyme (Collagenase HA; VitaCyte LLP, Indianapolis, IN) and either Neutral Protease (Serva Electrophoresis GMBH, Heidelberg, Germany) or Thermolysin (VitaCyte LLP, Indianapolis, IN) enzymes at 4 °C prior to warm dissociation at 37 **°**C in a recirculation loop. Iodixanol-based (Optiprep) continuous density gradient purification was performed after the collection of the digested pancreas, and islet fractions with ≥80% purity, ≥90% viability and a glucose-stimulated insulin secretion ratio of >1.0 were used for these studies. Human ICM (CMRL 1066, 10 mM Hepes, 2.5% human serum albumin, 10 ug/ml ciprofloxacin) was harvested from islet preparations (n = 10) after post-isolation culture for up to 72 hours, centrifuged at 1,200 × g for 15 min. to discard dead cells and debris, then stored at −80 °C until use. Relevant donor characteristics have been detailed in Supplementary Table S1, including the analyses of the islet preparations from each donor that were used to generate the ICM or ICL.

### Nanoparticle Tracking Analyses

Individual ICM samples, collected at 24 hours after isolation, were analyzed by the Nanosight NS500 system (Nanosight Ltd., Amesbury, UK). Human ICM were measured by NTA to quantify the mean size and concentration of particles. ICM preparations were diluted (1:50) in PBS and analyzed with the Nanoparticle Analysis System & NTA 1.4 Analytical Software. At least 5 recordings, 30 seconds each were obtained at 37 °C with a camera shutter speed set to 30.0 ms, a camera level of 14 and detection threshold set to 9.

### Flow cytometry Analyses

Flow cytometry analyses were performed on aliquots of ICM using a FACS Canto II with small particle analyzer, as previously described^13, 16, 26^. Samples of 25 µl ICM (n = 10), collected 24 hours after isolation, were labeled for 30 min. at RT with the following fluorescent-probe conjugated surface markers or antibodies: DAA anti-GAD65-PE (0.25 µg, clone 144, ImmuQuest), anti-IA-2-APC (0.4 µl, aa287–316, LifeSpan Biosciences), and anti-ZnT8-FITC (3 µg, aa263–369, LifeSpan Biosciences, Inc.); anti-Glut2-PE (5 µl, clone 199017, R&D Systems) and anti-HSC70-PE (2 µl, clone 1B5, LifeSpan Biosciences, Inc.); membrane vesicle or exosome markers Annexin V-V450 or -FITC (1 µl, BD Bioscience), CellTracker Deep Red-APC (10 µM, ThermoFisher Scientific), anti-CD9-V450 (2 µg, clone M-L13, BD Bioscience), anti-CD14-PE (10 µl, clone M5E2, BD Bioscience), and anti-Hsp70-APC (1 µg, clone C92F3A-5, LifeSpan Biosciences, Inc.). Details of the reagents used are outlined in Supplementary Table S2. EV gate was generated using green-fluorescent silica beads of 100, 500 and 1,000 nm (Kisker Biotech, Germany), and the EV counts and contour plots were obtained by gating on the Ag^+^AnnV^+^CT^+^EV population. Size estimation of the EV are thus based on the refractive index of the silica beads as measured at 488 nm. As such, the flow cytometry-based detection of EV reported fall between only slightly below the 100 nm beads to slightly above the 1,000 nm beads, and do not include EV much smaller the 100 nm silica beads. Background generated by the antibodies detected in PBS alone was subtracted from each value obtained. Values detected in the supplemented control, unconditioned media (from the same lots of media used to generate each individual ICM) were further subtracted from values detected in each ICM to ensure EV detected were in fact produced by the cultured islets. Additionally, controls were performed on ICM from four donors (n = 4) to ensure that genuine EV were detected and antibody specificity confirmed. These included analysis of the supernatant generated after each ICM was centrifuged at 100,000 g for 1 hour, ICM incubated with 50 mM EDTA or 0.3% Triton X-100 for 30 min., and compared to untreated ICM from the same donor, to deplete EV, prevent AnnV from binding to PS, and destroy membrane-bound vesicles, respectively.

### EV Enrichment

Vesicles were enriched from human ICM collected at 24 hours, using a protocol described previously^13^. Briefly, aliquots of 50 ml were thawed and fractionated by serial centrifugation at 50,000 × g for 15 min to isolate apoptotic body-like and MV-like EV, followed by 200,000 × g for 18 hours to isolate Exo-like EV, producing the ICM-50 K and ICM-200 K pellets, respectively, and stored at −80 °C until use.

### Mass Spectrometry

For proteomic analyses by LC-MS/MS, EV fractions were isolated from ICM from different donors (n = 5) through sequential centrifugation, as outlined above. Twenty micrograms of protein from fractions of ICM-50 K and ICM-200 K were separated by SDS-PAGE. Tryptic digests were analyzed by LC-MS/MS on an LTQ-Orbitrap Elite and the data were processed using PEAKS 7.0 (Bio-informatics Solutions), and the human Uniprot database, as previously described^13^. Processed data was compared to that of the ICL, and proteins identified in the ultracentrifuged 50 K and 200 K fractions derived from control, unconditioned media were subtracted from those detected in the respective fractions of ICM-50 K and ICM-200 K. Data analysis was performed with Scaffold 4.4 with a False Discovery Rate of 1%. Gene Ontology enrichment analysis for cellular component, molecular function and biological pathways were performed using FunRich v3.0, and only the enriched terms with a p-value of <0.01 were considered, as previously described^62^.

### Whole Mount Negative Staining and Immunogold Transmission Electron Microscopy

Whole mount negative staining and immunogold labeling experiments for TEM were performed on the aforementioned fractionated human ICM samples, as previously described^13, 63^. Briefly, a 1:1 dilution of the ICM-50 K or ICM-200 K pellets was created using a buffer of 4% paraformaldehyde in 50 mM HEPES, pH 7.4, and incubated for 1 hour. Aliquots of 10 µl were adsorbed to Formvar carbon-coated copper grids for 20 min. and contrasted for whole mount negative staining electron microscopy or immunolabeling. Immunogold double labeling was performed to confirm the results obtained by flow cytometry. Primary antibodies used were of the same clonality described above. After blocking with 1% bovine casein ovalbumin for 5 min., combinations of either anti-human GAD65, anti-human Glut2 or anti-human ZnT8 (epitope: aa263–369; LifeSpan Biosciences, Inc.) (clones as above) with anti-annexin V (R&D Systems) were incubated for 1 hour. Control samples were run in parallel by incubation for 1 hour with 1% bovine casein ovalbumin alone. Grids were incubated with a goat anti-mouse IgG conjugated to 18 nm colloidal gold (1:20 dilution; Jackson Laboratories) and goat anti-rabbit IgG conjugated to 12 nm colloidal gold (1:20 dilution; Jackson Laboratories) secondary antibodies for 30 min., followed by washing and fixation. Negative staining was performed with 10 µl of uranyl acetate for 40 sec., and then the grids were air dried for at least 30 min. prior to analyses on a FEI Tecnai 12 BioTwin 120 kV TEM. At least 50 fields of view were examined for the presence of gold particles at 13,000× magnification from each of the control and primary antibody-labeled grids. Electron micrograph images were captured on the Advanced Microscopy Techniques (AMT) XR80C CCD Camera System with AMT Image Capture Engine V601 at 68,000×, 49,000×, or 18,500× magnification.

### Effects of Pan-Caspase Inhibition on Human Islet EV Production

To examine the effect of the pan-caspase inhibitor ZVAD-fmk (ZVAD) on EV production, islets were cultured with media supplemented with 50 µM ZVAD, DMSO vehicle control (DMSO), or no intervention (Normal) at 37 °C for up to 72 hours. ICM was collected at 24, 48 and 72 hours, as outlined above, and stored at −80 °C until analysis by flow cytometry.

### Statistical Analyses

All data are expressed as means ± SEM derived from at least three independent experiments unless otherwise specified. Statistical significance of results was evaluated on raw values prior to conversion to percent of control, by a repeated measures one-way analysis of variance (ANOVA) test with Tukey’s post-hoc pairwise comparison, or t-test, using SPSS Statistics (Version 21). P values < 0.05 were considered significant.

## Electronic supplementary material

Supplementary Information
