# Supplementary material for: Presence of diabetes autoantigens in extracellular vesicles derived from human islets
Source: Sci Rep. 2017 Jul 10;7:5000. doi: 10.1038/s41598-017-04977-y (PMC5504025; doi:10.1038/s41598-017-04977-y)
Supplement: Supplementary file 1 — Supplementary Information [file 41598_2017_4977_MOESM1_ESM.docx]

**Supplementary Information**

**Presence of diabetes autoantigens in extracellular vesicles derived from human islets**

Craig P. Hasilo^1,2,5^, Sarita Negi^1,2,5^, Isabelle Allaeys^3^, Nathalie Cloutier^3^, Alissa K. Rutman^1,2,5^, Marco Gasparrini^1,2^, Éric Bonneil^4^, Pierre Thibault^4,5^, Éric Boilard^3,5^,Steven Paraskevas^1,2,5*^

^1^Human Islet Transplant Laboratory, McGill University Health Centre, Montréal, Québec, Canada, ^2^Research Institute of the McGill University Health Centre, Montréal, Québec, Canada, ^3^Centre de Recherche en Rhumatologie et Immunologie, Centre de Recherche du Centre Hospitalier Universitaire de Québec, Faculté de Médecine de l’Université Laval, Québec, Québec, Canada, ^4^Institut de Recherche en Immunologie et en Cancérologie, Université de Montréal, Montréal, Québec, Canada, ^5^Canadian National Transplant Research Program.

*Corresponding Author:

Steven Paraskevas, MD, PhD

Associate Professor of Surgery

Director, Pancreas and Islet Transplant Program

McGill University Health Centre

Royal Victoria Hospital

D5.5736, 1001 Decarie Blvd.

Montreal, Quebec, Canada H4A 3J1

[steven.paraskevas@mcgill.ca](mailto:steven.paraskevas@mcgill.ca)

Tel: (514) 934-1934 ext. 31951

Fax: (514) 843-1503

www.isletlab.org

**Supplementary Figure S1. Optimization of the detergent sensitivity of extracellular vesicles in islet conditioned media.** Bar graph of flow cytometry analysis showing a dose-dependent reduction in AnnV^+^EV detected up to a concentration of 0.3% Triton-X100.

**Supplementary Figure S2.** **Comparison of proteomic data by enrichment analyses of fractionated human islet conditioned media extracellular vesicles with FunRich molecular function.** Bar graphs illustrating the molecular function overrepresented in highly abundant proteins identified in islet conditioned media (ICM) ICM-50K (a) and ICM-200K (b).

**Supplementary Figure S3. Common markers of extracellular vesicles are present on islet-derived extracellular vesicles.** Bar graphs of untreated islet conditioned media (Control) compared to signal depletion by 100,000 x g centrifugation, 50 mM EDTA, or 0.3% Triton X-100 for CD9^+^AnnV^+^CT^+^EV (a), Hsp70^+^AnnV^+^CT^+^EV (b), and CD14^+^AnnV^+^CT^+^EV (c). Data are expressed as mean ± SEM from n=4 human islet preparations. *p<0.05, repeated measures one-way analysis of variance with Tukey’s post-hoc test performed on raw values prior to conversion to percent of control.

**Supplementary Figure S4. Validation of flow cytometry analyses of autoantigen specificity in human islet conditioned media-derived extracellular vesicles.** Forward- by side-scatter profiles were obtained by flow cytometry using silica beads of 100, 500 and 1,000 nm for size calibration on a FACS Canto II modified with a FSC-PMT-H small particles option (a). Islet conditioned media (ICM) were stained with both Annexin V (AnnV) and CellTracker Deep Red (CT) (b), or with AnnV, CT and anti-GAD65 (c). Representative contour plots showing the peak density of AnnV^+^CT^+^EV (b) and GAD65^+^AnnV^+^CT^+^EV (c) were detected in control, non-treated, ICM (upper panel) and compared to ICM subjected to 100,000 x g centrifugation (100,000 g), treatment with 50 mM EDTA (EDTA), or 0.3% Triton-X100 (Triton). Data are representative of results from n=4 human islet preparations.


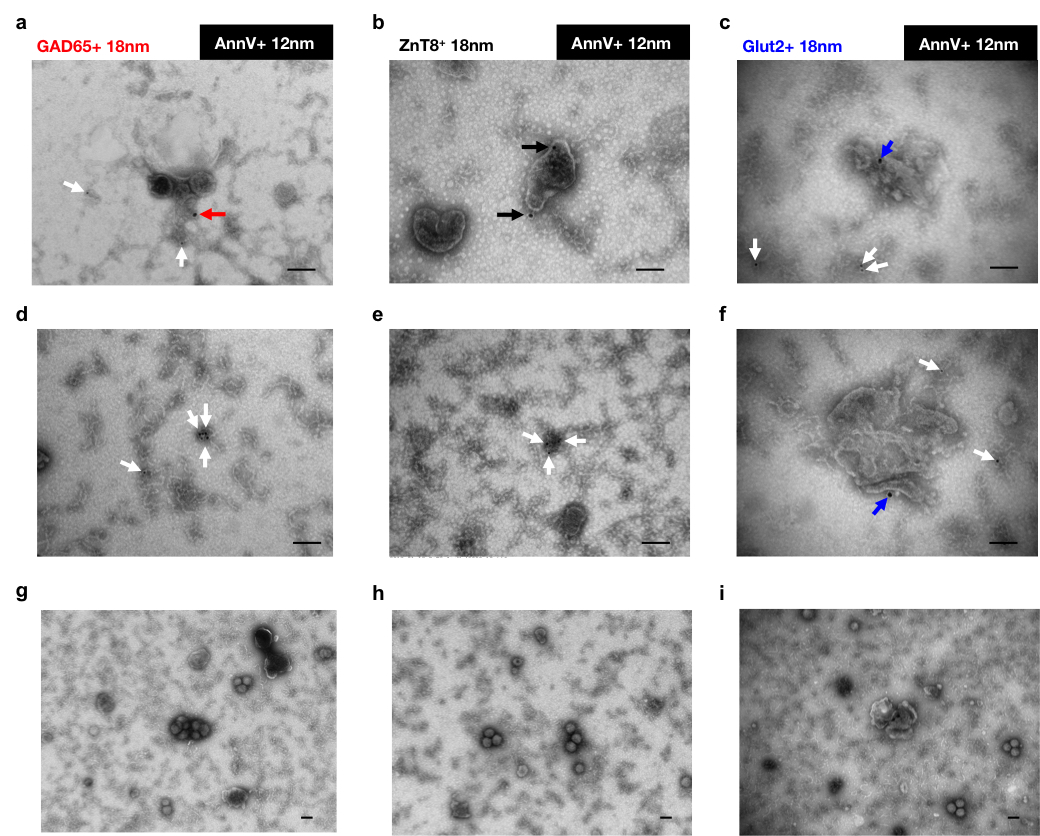


**Supplementary** **Figure S5. Presence of immuno-gold particle labeling of diabetes autoantigens GAD65 and ZnT8, and β-cell marker Glut2 on extracellular vesicles but not on vesicles in control samples.** Electron micrographs of immunogold double labeled islet-derived EV preparations from fractionated ICM confirms the presence of AnnV (a,c,d,e,f; white arrows), GAD65 (a; red arrows), ZnT8 (b; black arrow) and Glut2 (c,f; blue arrow). Grids incubated with control sera alone contain no gold particles (g,h,i). Scale bars represent 100 nm at 49,000 x (a-f) and 18,500 x magnification (g-i). Data are representative of n=3 independent experiments.


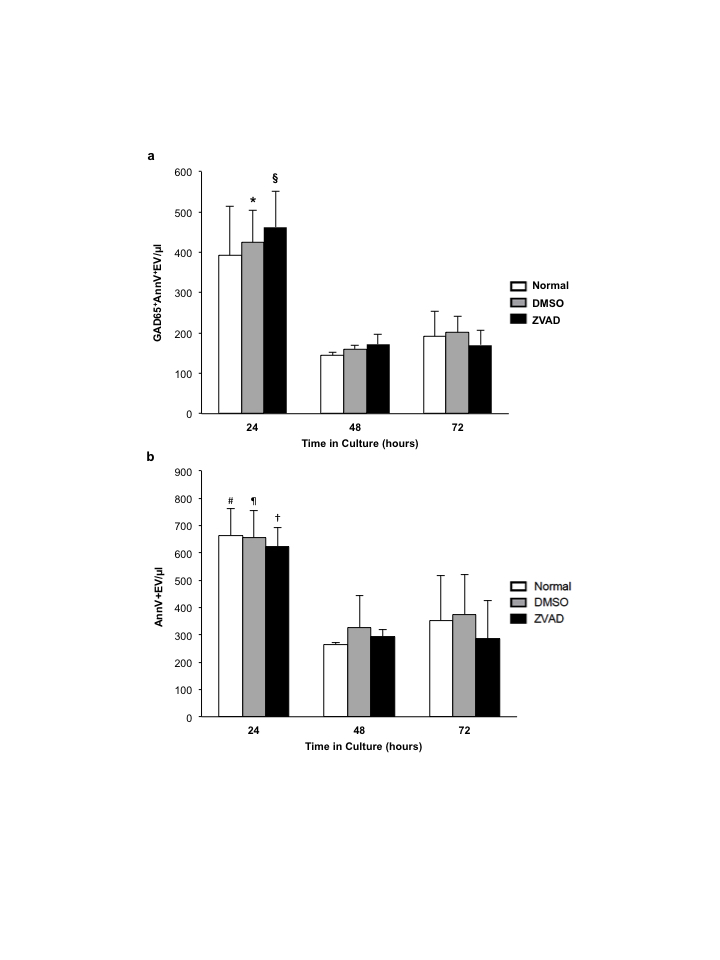


**Supplementary Figure S6. Islet-produced extracellular vesicles are eluted in a caspase-independent process over 72 hours in culture.** Bar graphs represent flow cytometry-based quantification of GAD65^+^AnnV^+^CT^+^EV (a) and AnnV^+^CT^+^EV (b) produced after treatment of cultured human islets with 50 µM pan-caspase inhibitor ZVAD-fmk (ZVAD, black bars), DMSO vehicle control (grey bars), or non-treated islets (Normal, white bars). Data are expressed as mean ± SEM from n=3 different donors islet preparations from n=3 independent experiments, *p<0.04 vs. 48 and 72 hours, §p<0.02 vs. 48 and 72 hours, # p<0.02 vs. 48 hours, ¶ p<0.02, p<0.05 vs. 48 hours, † p<0.05 vs. 48 and 72 hours, t-test.


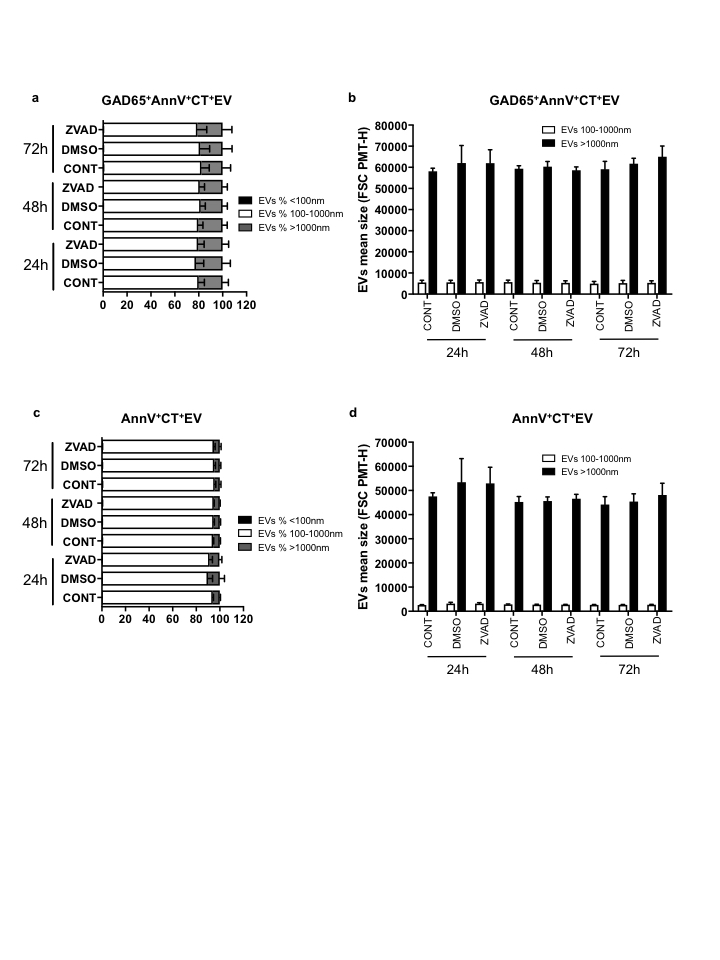
 **Supplementary Figure S7. Caspase inhibition does not effect the size of islet-produced extracellular vesicles over 72 hours in culture.** Bar graphs represent flow cytometry-based quantification of GAD65^+^AnnV^+^CT^+^EV (a,b) and AnnV^+^CT^+^EV (c,d) produced after treatment of cultured human islets with 50 µM pan-caspase inhibitor ZVAD-fmk (ZVAD), vehicle control (DMSO), or non-treated islets (CONT). Comparison of percent GAD65^+^AnnV^+^CT^+^EV (a) and AnnV^+^CT^+^EV (c) that are <100 nm (black bars), 100-1,000 nm (white bars) and >1,000 nm (grey bars) in size at 24, 48 and 72 hours. (b) Comparison of mean FSC PMT-H of GAD65^+^AnnV^+^CT^+^EV (b) and AnnV^+^CT^+^EV (d) between 100-1,000 nm (white bars) and >1,000 nm (black bars) at 24, 48 and 72 hours. Data are expressed as mean ± SEM, n=3 donor islet preparations from n=3 independent experiments.

**Supplementary Table S1. Donor characteristics and islet assessment performed to generate ICM from each of the ten different donors (n=10).**

|  | Donor Characteristics | | | | | Islet Assessment | | | |
| --- | --- | --- | --- | --- | --- | --- | --- | --- | --- |
|  | Sex | COD^*^ | Age (years) | BMI^#^ | CIT^§^ (hours) | Purity (%) | Initial Viability (%) | Final Viability (%) | Static GSIS^♮^ (ratio) |
|  | Male | Cerebral hemorrhage | 46 | 23.3 | 11.2 | 90.0 | 97.0 | 97.1 | 3.7 |
|  | Male | Cerebral vascular accident | 60 | 26.0 | 7.0 | 95.0 | 95.5 | 95.5 | 4.1 |
|  | Female | Cerebral vascular accident | 65 | 23.9 | 6.5 | 80.0 | 92.3 | 85.7 | 12.1 |
|  | Male | Subarachnoid hemorrhage | 48 | 30.5 | 2.7 | 93.5 | 96.8 | 96.8 | 3.1 |
|  | Female | Subarachnoid hemorrhage | 64 | 28.6 | 11.8 | 80.0 | 95.0 | 95.0 | 4.0 |
|  | Male | Cranial trauma | 46 | 32.4 | 6.1 | 85.0 | 98.0 | 98.0 | 2.1 |
|  | Male | Cranial anoxia | 41 | 23.9 | 7.5 | 87.5 | 95.5 | 95.5 | 1.3 |
|  | Male | Cranial trauma | 53 | 29.3 | 8.8 | 80.7 | 92.0 | 96.0 | 1.8 |
|  | Male | Cranial trauma | 44 | 22.8 | 5.1 | 96.5 | 98.0 | 98.0 | 2.2 |
|  | Male | Cranial trauma | 47 | 26.1 | 4.2 | 90.0 | 96.0 | 96.0 | 9.8 |
| Mean±SEM | 8 M/2 F |  | 51.4  ±2.7 | 26.7  ±1.1 | 7.1  ±0.9 | 87.8  ±2.0 | 95.6  ±0.7 | 95.4  ±1.1 | 4.4  ±1.1 |

^*^COD, cause of death; ^#^BMI, body mass index; ^§^CIT, cold ischemia time; ^♮^GSIS, glucose-stimulated insulin secretion.

**Supplementary Table S2. Flow cytometry reagents and their working quantities.**

| **Target Antigen** | **Clonality** | **Amount per test** | **Dilution** | **Fluorophore** | **Product ID** |
| --- | --- | --- | --- | --- | --- |
| CD9 | Monoclonal | 2 μg | 1:3 | V450 | BD Biosciences, 341647 |
| AnnV |  | 2 μl | 1:125 | V450 | BD Biosciences, 560506 |
| AnnV |  | 2 μl | 1:125 | FITC | BD Biosciences, 556420 |
| ZnT8 | Polyclonal | 3 μg | 1:17 | FITC | LifeSpan BioSciences, Inc., LS-C303154 |
| GAD65 | Monoclonal | 0.25 μg | 1:1000 | PE | ImmuQuest, IQ442RPE |
| Glut2 | Monoclonal | 5 μl | 1:50 | PE | R&D Systems, FAB1414P |
| CD14 | Monoclonal | 10 μl | 1:25 | PE | BD Biosciences, 555398 |
| Glutathione-S transferase (IgG Control) | Monoclonal | 0.5 μg | 1:500 | PE | ImmuQuest, IQ442RPE |
| Hsp70 | Monoclonal | 1μg | 1:500 | APC | LifeSpan BioSciences, Inc., LS-C231813 |
| Hsc70 | Monoclonal | 2 μl | 1:125 | APC | LifeSpan BioSciences, Inc., LS-C231273 |
| IA-2 (PTPRN) | Monoclonal | 0.4 μl | 1:600 | APC | LifeSpan BioSciences, Inc., LS-C284886 |
| CellTracker Deep Red |  | 10 μM |  | APC | Life Technologies Inc., C34565 |
